# Twentieth Century Practice of Medicine

**Published:** 1896-07

**Authors:** 


### Book Notices.

---

TWENTIETH CENTURY PRACTICE. An International Encyclopedia of Modern Medical Science. By Leading Authors of Europe and America. Edited by Thomas L. Stedman, M. D., New York City. In Twenty Volumes. Volume V. "Diseases of the Skin." New York: William Wood & Co. 1896.

It is with pleasure that we announce to our readers the publication of volume V. of the Twentieth Century Practice. This volume contains more than nine hundred pages, and treats entirely of diseases of the skin. The contributors to it are a number of the leading dermatologists of the world—among them being Dr. Chas. W. Allen, of New York; Dr. John T. Bowen, of Boston; Dr. L. Brocq, of Paris; Dr. L. Duncan Bulkley, of New York; Dr. H. Radcliffe Crocker, of London; Dr. James Nevins Hyde, of Chicago; Dr. Moriz Kaposi, of Vienna; Dr. H. Leloir, of Lille, France; Dr. Douglass W. Montgomery, of San Francisco; Dr. Arthur Van Harlingen, of Philadelphia; and Dr. Henry H. Whitehouse, of New York. As would be expected from such a list of contributors, this volume covers the entire range of diseases of the skin in an exhaustive, yet concise and practical way. No branch of medicine is more important than the one here considered, and with all the splendid volumes which have preceded it, none are better or of more worth to the physician than this one. H.

